# Construction of root tip density function and root water uptake characteristics in alpine meadows

**DOI:** 10.3389/fpls.2022.918397

**Published:** 2022-10-24

**Authors:** Bin Deng, Baisha Weng, Denghua Yan, Shangbin Xiao, Haotian Fang, Meng Li, Hao Wang

**Affiliations:** ^1^ State Key Laboratory of Simulation and Regulation of Water Cycle in River Basin, China Institute of Water Resources and Hydropower Research, Beijing, China; ^2^ Engineering Research Center of Eco-Environment in TGR Region, Ministry of Education, College of Hydraulic and Environmental Engineering, China Three Gorges University, Yichang, China; ^3^ Yinshanbeilu National Field Research Station of Steppe Eco-Hydrological System, China Institute of Water Resources and Hydropower Research, Hohhot, China; ^4^ Department of Hydraulic Engineering, Tsinghua University, Beijing, China

**Keywords:** alpine meadow, root tip, root tip density equation, root water uptake model, regularity of root water uptake

## Abstract

Accurate calculation of root water uptake (RWU) is the key to improving vegetation water use efficiency and identifying water cycle evolution patterns, and root tips play an important role in RWU. However, most of the current RWU models in the alpine meadow are calculated based on the root length density (RLD) function. In this study, a large number of roots, soil hydraulic conductivity, and physicochemical property indices were obtained by continuous field prototype observation experiments for up to 2 years. It was found that the RLD and root tip density (RTD) in alpine meadows decrease by 16.2% and 14.6%, respectively, in the wilting stage compared to the regreening stage. The RTD distribution function of the alpine meadow was constructed, and the RWU model was established accordingly. The results show that the RTD function is more accurate than the RLD function to reflect the RWU pattern. Compared with RLD, the simulated RWU model constructed by using RTD as the root index that can effectively absorb water increased by 24.64% on average, and the simulated values were more consistent with the actual situation. It can be seen that there is an underestimation of RWU calculated based on the RLD function, which leads to an underestimation of the effect of climate warming on evapotranspiration. The simulation results of the RWU model based on RTD showed that the RWU rate in the regreening stage increased by 30.24% on average compared with that in the wilting stage. Meanwhile, the top 67% of the rhizosphere was responsible for 86.76% of the total RWU on average. This study contributes to the understanding of the alpine meadow water cycle system and provides theoretical support for the implementation of alpine meadow vegetation protection and restoration projects.

## 1 Introduction

Alpine meadows are a type of vegetation ecosystem unique to the Qinghai-Tibet Plateau (QTP). In the context of climate warming, the climatic characteristics of the QTP have been significantly affected (Yuke, 2019). The alpine meadow also shows a clear trend of degradation (Ma et al., 2017) due to its extremely fragile ecosystem (Jin et al., 2019). The key to plant growth is the ability to utilize water, and an accurate grasp of the water uptake capacity of plants will help to protect and restore the alpine meadow ecosystem reasonably. Meanwhile, plants and their roots are also an important part of the water cycle (Wu et al., 1999). Plants directly influence the distribution of soil water through root uptake (Liao et al., 2018), and the vast majority of the absorbed water is released to the atmosphere in the form of canopy transpiration in addition to its growing consumption, connecting soil water to the atmospheric environment (Hupet et al., 2002). Therefore, an accurate calculation of the amount of water absorbed by roots is essential to improving the efficiency of plant water use and also to helping in the identification of the evolutionary pattern of the water cycle (Metselaar and Lier, 2011).

A common approach to quantifying the rate of RWU is to build an RWU model for simulation (Ojha et al., 2009; Barkaoui et al., 2016); usually, a one-dimensional macroscopic RWU model is used (Cowan, 1965; Janott et al., 2011). The external factors that affect water uptake by plants are soil moisture and meteorological conditions (Lai and Katul, 2000; Hodge et al., 2009), and as the soil acts on RWU (Jha et al., 2017), the water content of the soil near the root zone is likewise changing (Jia et al., 2016). Considering the factors affecting soil moisture changes, it is necessary to analyze the influence of the soil’s water dynamic characteristics on RWU (Guo et al., 2019; Zhang and Huang, 2021). The role of soil hydraulic conductivity as a key index of the changes produced by soil water dynamics (Lebron et al., 2007) cannot be ignored. Therefore, the characteristics of RWU distribution described by the Selim–Iskandar model (Fred and Molz, 1981), which combines root indicators with soil hydraulic conductivity, may be more accurate. The plant’s factor that affects its water uptake capacity is the density of the root index that can effectively absorb water. Driven by the growth and transpiration demand of plants (Peter et al., 2018), plants absorb soil water through the action of the roots, and the density of the root index that can effectively absorb water determines the water uptake efficiency of the roots. Therefore, an accurate grasp of the root index that can effectively absorb water is the key to the simulation process.

Nowadays, the methods for simulating the RWU rate that includes root parameters are based on RLD as the root index that can effectively absorb water (Molz and Remson, 1970). In the past, it was difficult to measure the root index that can effectively absorb water, so an exponential type of RWU model was established using the RLD distribution function, which is simpler to measure as the root index that can effectively absorb water (Li et al., 1999), which has greatly improved the progress of the study of RWU models. The models widely used today are based on this type of approach and have been used extensively in the study of field crops and trees (Faria et al., 2010; dos Santos et al., 2016; Su et al., 2017). However, these models may have some errors in the simulation process due to the inaccurate description of the root index that can effectively absorb water.

It has been shown that the main sites of water uptake in plants are the hairy roots and root tips of the roots (Gilroy and Jones, 2000; Luo et al., 2000; Huang et al., 2020). Different areas on the root segments have different water permeabilities (Nancy and Ian, 2012), with hairy roots and root tips exhibiting higher permeability. Their growth expands the root index that can effectively absorb water (Segal et al., 2008), ensuring that the roots can absorb sufficient water from the soil for their own growth needs and respiratory consumption. Therefore, root tips play an important role in RWU, and RTD determines the water uptake capacity of the roots. Thus, this paper accurately measured the RTD distribution characteristics by the minirhizotron technique and used it as the root index that can effectively absorb water to assess the water uptake characteristics of plants. The established model is more consistent with the actual physical laws, and its simulation performance may be improved to a certain extent compared with the traditional model.

**Figure 1:**
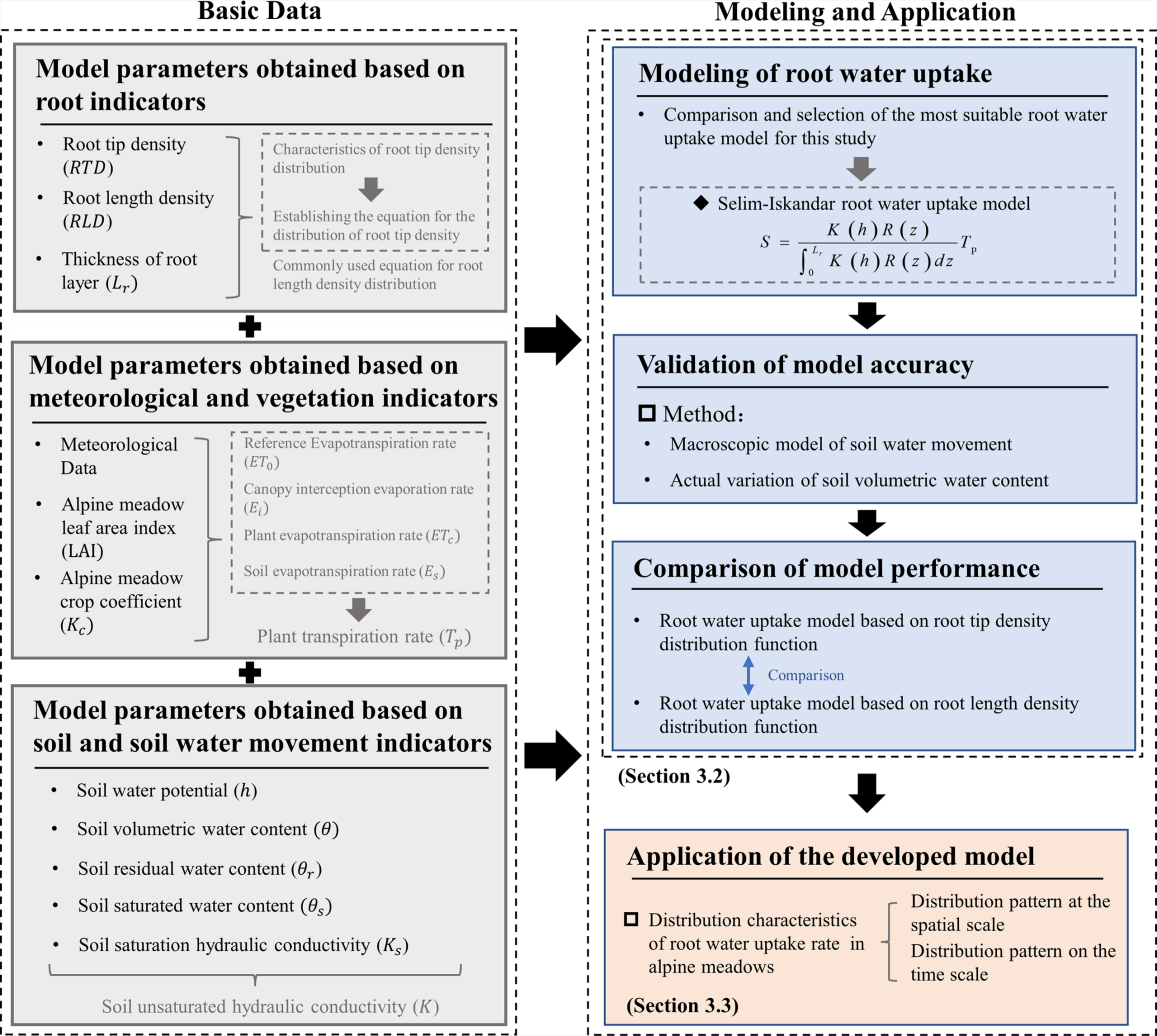
Research process.

This study aims to identify the RWU pattern of alpine meadows and solve the problem of estimating the water consumption capacity of alpine meadow vegetation and its RWU simulation method. The flow of the study is shown in **Figure 1**. The functional equation of root tip distribution of alpine meadows is constructed with the meadow roots as the research object, and the accuracy of the soil water change state reflected by the model is evaluated when the RTD function and RLD function are used as the root index that can effectively absorb water of the model based on Selim–Iskandar’s RWU model. To eliminate the effects caused by experimental errors, the model was validated using measured soil water content data from Exp. 1~4 at four experimental sites a, b, c, and d on the QTP. The established RWU model was used to simulate and analyze the RWU patterns of alpine meadows at different phenological stages and altitudes. The improved simulation method in this study will help the development of the RWU model and the simulation results will provide theoretical support for the study of vegetation ecohydrological cycle in alpine meadows and the implementation of vegetation restoration projects.

## 2 Materials and methods

### 2.1 Experimental sites

The study area is located in the hinterland of QTP (30°54′~32°43′N, 91°12′~92°54′E), and the experimental sites are shown in **Figure 1** and **Table 1**. The climate type is a typical semiarid monsoon climate of the plateau subduction zone, which generally shows thin air, abundant sunshine, and strong radiation, with an annual average sunshine time of 2,723 h. The weather is cold and dry with a large temperature difference between day and night. The temperature decreases with the increase in altitude and latitude, with an annual average temperature of -0.6°C, a maximum temperature of 14.5°C, and a minimum temperature of -30°C. Rainfall is unevenly distributed during the year, with the warm and wet period from June to October being the peak period for rainfall, which accounts for 82.9% of the year (Lu, 2017; Gong, 2019). The entire QTP is rich in vegetation species, and the study area shows the most typical *Kobresia pygmaea*, covering more than 80% of the area. The vegetation is more fragile and degraded to some extent (Jiang et al., 2020; Duan et al., 2021). The study area contains a variety of soil types such as bog soils, alluvial soils, and felty soils, among which felty soils are the most abundant, accounting for 67.5% of the total area of the study area.

**Table 1:** Measured experimental sites according to altitude (H), geographical location; soil type (ST) of experimental sites in the watershed obtained using remote sensing images; crop coefficients with mean values (*k_c1_*) in the regreening stage and mean values (*k_c2_*) in the wilting stage; the average leaf area index (LAI) for each location was obtained from the “National Tibetan Plateau Scientific Data Center” (http://data.Tpdc.ac.cn) (Zhang, 2021).

Sites	Longitude	Latitude	H (m)	ST	*k_c1_*	*k_c2_*	LAI
a	91°58′34″	31°25′4″	4460	Alpine meadow soil	0.68	0.42	1.44
b	91°41′34″	31°3′52″	4730	Alpine meadow soil	0.65	0.4	2.32
c	91°35′9″	32°16′47″	4760	Alpine meadow soil	0.55	0.33	1.29
d	91°49′26″	32°33′15″	5050	Alpine meadow soil	0.51	0.28	1.02

### 2.2 Selection of experimental sites and installation of experimental equipment

**Figure 2:**
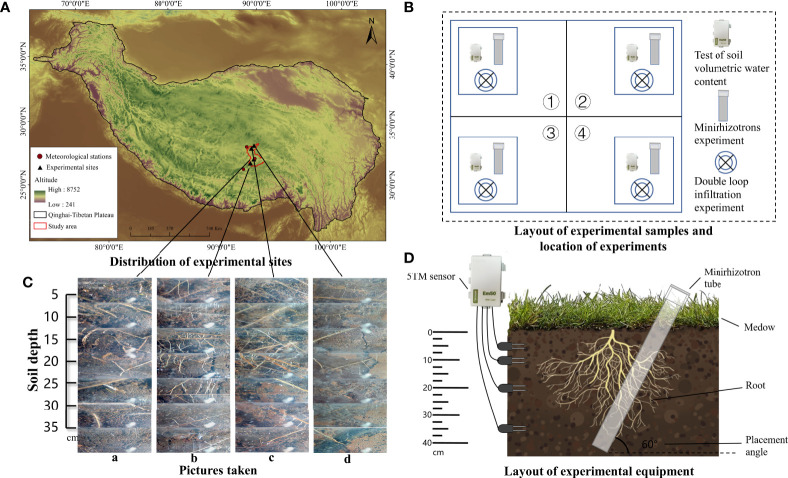
The distribution of experimental sites **(A)** and the meteorological stations selected for data are included, as well as the location of the study area and experimental sites. The layout of experimental samples and location of experiments **(B)** describes the distribution of the experimental sample at each experimental site, the location of each experiment. The pictures taken by the minirhizotron camera **(C)** and represent a set of photographs taken by the minirhizotron technique at sites a, b, c, and d at different soil depths, with significant variability in root density distribution at different experimental sites. The layout of experimental equipment **(D)** describes the method of deployment of the continuous soil water content and soil water potential testing instrument (5TM sensor) and minirhizotron tube.

The study area has unique geographical and climatic conditions typical of alpine regions. In alpine regions, altitude, soil water content, and soil hydraulic conductivity are three important environmental factors affecting root growth and water uptake capacity, which are also important criteria for the selection of the experimental sites in this study. To ensure that the location of the selected experimental sites can accurately reflect the overall vegetation and eco-hydrological characteristics of alpine meadows but also facilitate the safety of field experiments, after a long field study, we established four typical experimental sites in August 2018 (**Figure 2A**).

Four experimental sample sites with more consistent vegetation and soil conditions were selected before deployment. According to the root distribution characteristics of the alpine meadow, we dug out a deep pit of nearly 50 cm and placed soil water potential and moisture sensors (instrument type 5TM) at 5, 10, 20, and 35 cm each and then buried the pit with the original soil to ensure that the soil could recover to its native state as quickly as possible (**Figure 2D**).

For root measurements using the minirhizotron technique (**Figure 2D**), minirhizotron tubes were laid out using the soil coring method at each experimental site in a circular hole dug at a depth of approximately 1 m and 30° in the vertical direction within four sample squares. A polyvinyl chloride transparent minirhizotron tube (1-m length, 6.4-cm inner diameter, 7-cm outer diameter) was inserted into the holes (Johnson et al., 2001); the mouth of the tube was covered with a cap and sealed with a void to prevent water infiltration, which could form water droplets on the inner wall and affect the subsequent minirhizotron window photography.

The installation of micro-root tubes can cut the plant roots and disturb the original state of the soil (Joslin and Wolfe, 1999), and the placement of soil sensors can also cause changes in soil temperature and humidity. Therefore, a 2-year recovery period for vegetation and soil ecology was given to each experimental site, while the experimental equipment was regularly checked and maintained, and the experimental monitoring was started in 2020.

### 2.3 Experimental design

#### 2.3.1 Measurement of root indicators

Since this study focused on the RWU characteristics, the selection of the phenological stage was based on the phenological characteristics of the underground roots of alpine meadows. The beginning and end of the phenological period in the underground part of alpine meadows are the regreening and wilting stages. In the study area, the regreening stage is mainly concentrated in June and the wilting stage is concentrated in September each year. Considering this typical phenological feature, the experiment was conducted in June 2020 (Exp. 1), September 2020 (Exp. 2), June 2021 (Exp. 3), and September 2021 (Exp. 4).

The distribution of the experimental samples and the position of each experiment in the sample are shown in **Figure 2B**. We observed the morphological characteristics of plant roots through the minirhizotron technique [minirhizotron technique containing a minirhizotron tube, optical camera, calibration handle, controller, and computer (Ahrens and Reichstein, 2014)]. The camera was extended into the minirhizotron tube and moved down 5.8 cm at a time (about 5-cm vertical depth). The camera lens had a range of 2 cm × 2 cm and was rotated 45° after taking one photograph to ensure the integrity of the photograph. Eight consecutive photographs were taken and then moved down one layer until the roots could not be found in the lens. Based on this method, the minirhizotron tubes in the four sample squares of each experimental site were photographed (**Figure 2C**). It was found that almost no roots were found in the study area after 35 cm, so the thickness of the root layer was considered to be 35 cm (**Table 2**). The 896 root images taken were later processed by WinRHIZO TRON MF 2018b image analysis software to obtain root morphological data such as root tip number and root length. The RTD and RLD per unit soil volume were calculated by the formula:


(1) $$D_{RT}=\frac{T\text{ips}}{A.DOF}$$

(2) $$D_{RL}=\frac{L}{A.DOF}$$

**Table 2:** The soil indicators and infiltration indexes for experimental sites a, b, c, and d which were measured in Exp. 1, Exp. 2, Exp. 3, and Exp. 4 are described, containing the thickness of the root layer (*L_r_*); soil saturated hydraulic conductivity during the regreening and wilting stages (*K_s1_* and *K_s2_*); saturated soil water content (*θ_s_*) and residual soil water content (*θ _r_*); average soil water content in Exp. 1, Exp. 2, Exp. 3, and Exp. 4 (*θ*_1_, *θ*_2_, *θ*_3_, *θ*_4_); and *L*_1_,*L_2_*, and *L*_3_ for soil depths of 0–10, 10–20, and 20–35 cm, respectively.

	*L_r_* (cm)	*K_s1_* (cm.min^-1^)	*K_s2_* (cm.min^-1^)	*θ_s_* (%)	*θ_r_* (%)	*θ_1_* (%)			*θ_2_* (%)			*θ_3_* (%)			*θ_4_* (%)		
						*L_1_*	*L_2_*	*L_3_*	*L_1_*	*L_2_*	*L_3_*	*L_1_*	*L_2_*	*L_3_*	*L_1_*	*L_2_*	*L_3_*
a	35	0.012	0.013	38.62	12.17	27.51	28.24	22.38	21.72	14.14	17.05	24.3	28.5	27.3	22.45	26.09	20.48
b	35	0.099	0.17	32.64	7.43	14.82	14.03	12.94	11.85	12.66	11.32	17.92	27.66	9.78	19.03	15.74	12.39
c	35	0.095	0.076	40.41	15.87	21	23.19	27.64	17.01	20.48	23.11	20.72	20.37	23.92	20.79	20.3	24.75
d	35	0.068	0.057	28.27	7.41	14.96	22.31	21.75	16.03	10.4	10.83	20.64	24.38	22.53	16.41	24.06	22.29

where *D_RT_* is the RTD per unit soil volume (tips.cm^-3^); *D_RL_* is the RLD per unit soil volume (cm.cm^-3^); *L* is the total root length per layer observed by the minirhizotron camera (cm); *Tips* is the total number of root tips per soil layer observed by the minirhizotron camera; *A* is the area of the picture taken by the observation window (cm^2^); and *DOF* is the distance from the minirhizotron camera to the surrounding photographed soil, which was taken as 0.3 cm (Wu et al., 2014).

#### 2.3.2 Measurement of soil indicators and infiltration indicators

For the observation of root indicators, the water content and water potential values in the soil were obtained by reading the 5TM sensors in the sample cubes through the ECH2O Utility software, and the recording frequency was automatically recorded every 1 h. Soil water content (SWC) and soil water potential data for the regreening and wilting stages in 2020 and 2021 were used.

The double-ring infiltration experiments were conducted in four samples a, b, c, and d during the regreening and wilting stages in 2021 (Rnnqvist, 2018), and we obtained data on the saturated hydraulic conductivity of the soil at each experimental site in the study area during the regreening and wilting stages (**Table 2**). The experimental equipment was the DJ-IN12-W double-ring infiltrator, whose inner and outer ring diameters were 60 and 30 cm, respectively, to minimize the influence of soil spatial heterogeneity on the experiment (Li et al., 2019). The Mariotte tubes with volumes of 3 and 10 l were configured to supply water to the inner and outer rings, respectively.

Soil samples were collected at 5, 10, 20, and 35 cm using the soil coring method without destroying the minirhizotron tubes. Total organic matter, pH, total carbon, total nitrogen, total phosphorus, total salt, fast-acting potassium, saturated SWC, residual SWC, porosity, bulk weight, and agglomerate composition were measured in the laboratory according to international standards.

### 2.4 Construction and validation of a root water uptake model for alpine meadows

#### 2.4.1 Construction of a root uptake model

In this paper, the Selim–Iskandar model (Fred and Molz, 1981) was selected to simulate the RWU by alpine meadows:


(3) $$S=\frac{K\left(h\right)R\left(z\right)}{\int_{0}^{L_{r}}K\left(h\right)R\left(z\right)dz}T_{r}$$

where *S* is the RWU rate (mm.day^-1^); *K* (h) is the soil unsaturated hydraulic conductivity (cm.min^-1^); *R*(*z*) is the root density function that can effectively absorb water; *L_r_* is the thickness of the root layer (cm); *z* is the depth from the ground surface (cm); and *T_r_* is the plant transpiration rate (mm.day^-1^).

The plant transpiration rates were simulated as follows (Belmans et al., 1983; Allen et al., 1998; Montero et al., 2001; Rouphael and Colla, 2004; Allen et al., 2006; Ojha et al., 2009; Fan, 2011; Genxu et al., 2012):


(4) $$ET_{c}=K_{c}\times ET_{0}$$

(5) $$ET_{0}=\frac{0.408\Delta \left(R_{N}-G\right)+\gamma \frac{900}{T+273}U_{2}\left(e_{s}-e_{a}\right)}{\Delta +\gamma \left(1+0.34U_{2}\right)}$$

(6) $$E_{i}=0.0025\times F_{c}\times Ri^{0.34}\times T^{0.19}$$

(7) $$E_{s}=ET_{c}.f.e^{-c.LAI}$$

(8) $$T_{r}=ET_{c}-E_{s}-E_{i}$$

where *ET_c_* is the plant evapotranspiration rate (mm.day^-1^); *ET*_0_ is the vegetation reference evapotranspiration rate (mm.day^-1^); *k_c_* is the crop coefficient **(** **Table 1**); *G* is the sensible heat flux density from the surface to the soil (MJ.m^-2^.d^-1^); *R_N_* is the net radiation (MJ.m^-2^.d^-1^) of vegetation; *U*_2_ is the wind speed (m.s^-1^) at the observed altitude; *e_s_* is the saturation vapor pressure (kPa); *e_a_* is the actual vapor pressure (kPa); *Δ* is the slope of the saturation vapor pressure versus temperature curve (kPa.°C^-1^); *γ* is the humidity constant (kPa.°C^-1^); *T* means the mean air temperature (°C) at the observed altitude; *E_i_* is the canopy interception evaporation rate (mm.day^-1^); *F_c_* is the vegetation cover; *R_i_* is the rainfall rate (mm.h^-1^); *E_s_* is the soil evapotranspiration rate; and *f* and *c* are regression coefficients. The plant transpiration rate at each experimental site is shown in **Table 3**.

**Table 3:** Plant transpiration rate at each experimental stage.

Sites	*T_p_* (mm.day^-1^)			
	Exp. 1	Exp. 2	Exp. 3	Exp. 4
a	1.92	1.60	2.16	1.89
b	2.04	1.73	2.29	1.97
c	1.87	1.54	2.14	1.87
d	1.81	1.49	2.03	1.71

The soil unsaturated hydraulic conductivity was simulated as follows (Mualem, 1976; van Genuchten, 1980):


(9) $$S_{e}=\frac{\theta -\theta_{r}}{\theta_{s}-\theta_{r}}={\left[1+{\left(\eta h\right)}^{n}\right]}^{\frac{1}{n}-1}$$

(10) $$K\left(h\right)=K_{s}S_{e}^{0.5}{\left[1-{\left(1-S_{e}^{\frac{1}{m}}\right)}^{m}\right]}^{2}$$

where *S_e_* is the effective soil saturation; *θ* is the measured soil water content (%) (**Table 2**); *θ*_s_ is the saturated soil water content (%);*θ*_r_ is the residual soil water content (%); *h* is the soil water potential (m); and *n* are empirical constants. Combined with the measured values, we obtained *n* and *n* for the individual experimental site by the inverse method.

The detailed calculation process and the acquisition method of each parameter are described in the **Appendix**.

#### 2.4.2 Validation method of the root water uptake model

A macroscopic model of soil water movement considering RWU (Shao et al., 1986) can be tested for the RWU model, with the model equation:


(11) $$\frac{\partial \theta }{\partial t}=\frac{\partial }{\partial z}\left[D\left(\theta \right)\frac{\partial \theta }{\partial z}\right]-\frac{\partial K\left(\theta \right)}{\partial z}-S\left(z,t\right)$$

where t is time; *D*(θ) is the soil water diffusion rate; *K*(θ ) is the unsaturated soil hydraulic conductivity; and *S*(*z,t*) is the RWU term. A discretization of the equation yields:


(12) $$\frac{\theta_{i}^{j+1}-\theta_{i-1}^{j+1}}{\Delta t}=\frac{D_{i+\frac{1}{2}}^{j+1}(\theta_{i+1}^{j+1}-\theta_{i}^{j+1})-D_{i-\frac{1}{2}}^{j+1}(\theta_{i}^{j+1}-\theta_{i-1}^{j+1})}{{\left(\Delta z\right)}^{2}}-\frac{\left(K_{i+1}^{j+1}+K_{i}^{j+1})-(K_{i}^{j+1}+K_{i-1}^{j+1}\right)}{2\Delta z}-S_{i}^{j+\frac{1}{2}}$$

The 35-cm soil layer is divided into seven layers equally, and the node number is defined as i. The spatial step Δz = 5 cm is set, the time step Δt = 1 day, and the node number is j. The initial conditions and boundaries are:


(13) $$\{\begin{array}{cc} \theta (z,0)=\theta_{0}(z) & 0\leq z\leq L_{r},t=0\\ -D\left(\theta \right)\frac{\partial \theta }{\partial z}+K\left(\theta \right)=-E_{s} & z=0,t>0\\ \theta (L_{r},t)=\theta_{L_{r}}(t) & z=L_{r},t>0 \end{array}$$

For the solution of the model, we calculate the soil water content on different soil layers based on the measured soil water content by equation recursion combined with numerical iterations.

In this paper, the root mean square error (RMSE) is used to assess the agreement between the simulation results of the model and the measured data:


(14) $$RMSE=\sqrt{\frac{1}{n}\sum_{i=1}^{n}{\left(S_{i}-M_{i}\right)}^{2}}$$

where n is the number of measured data; *S_i_* is the simulated value; and *M_i_* is the measured value.

## 3 Results

### 3.1 Characteristics of alpine meadow root distribution and construction of distribution equation

#### 3.1.1 Characteristics of root distribution in alpine meadows

**Figure 3:**
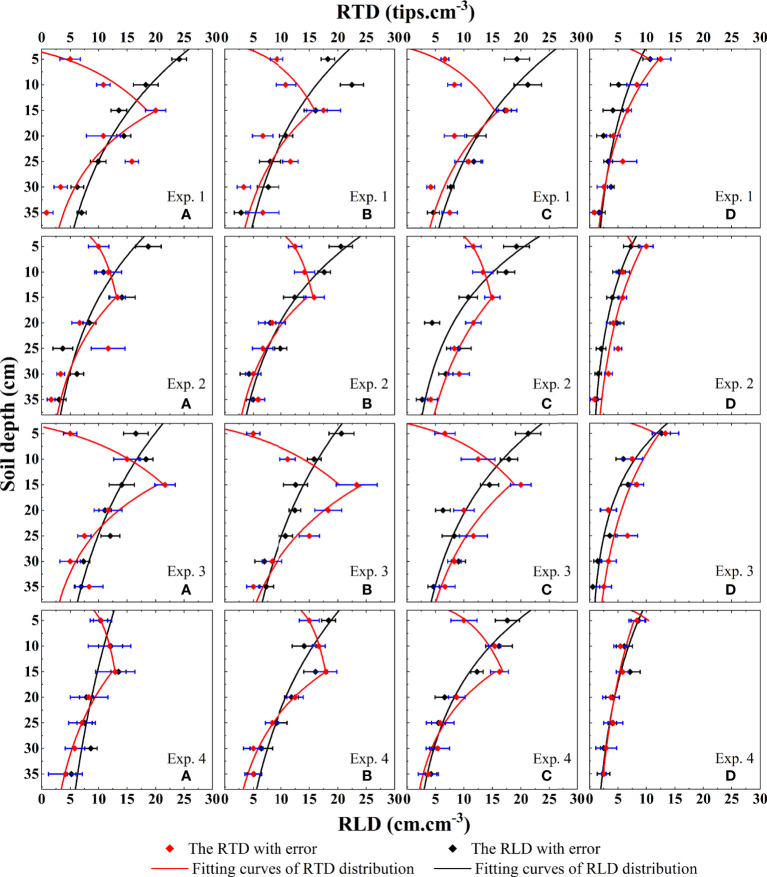
Based on the distribution characteristics of alpine meadow root indicators on soil profiles obtained from field experiments. The alpine meadow root length density index and root tip density index were included. The distribution equations of root tip density and root length density on the soil profile were fitted by Eq 15 and Eq 16 at the sites a, b, c, and d in Exp. 1 ~ 4.

The distribution of root indexes in alpine meadows is shown in **Figure 3**, and it shows a certain regularity in time and space. In terms of time, comparing the root density at the regreening stage and the wilting stage, we can find that the root density at the regreening stage of the alpine meadow is significantly greater than that at the wilting stage. Compared with the regreening stage, the RTD decreased by 14.6% and the RLD decreased by 16.2% on average in the wilting stage.

Spatially, from the lowest altitude a to the highest altitude d, both RTD and RLD showed a trend of increasing and then decreasing, and within the soil, there was a clear difference between the two root density indicators. At a, b, and c below the 5,000-m altitude, the maximum values of RTD were mainly concentrated in the middle layer of the rhizosphere, while the maximum values of RLD were mainly concentrated in the shallow layer of the rhizosphere. At d above 5,000 m, the distribution characteristics of RTD and RLD were very similar, both showing a trend of decreasing from shallow to deep.

#### 3.1.2 Construction of root tip density equation and simulation of root distribution

There are obvious regularities in the distribution of RTD on the soil profile in alpine meadows. Therefore, in this paper, a generalized equation Eq 15 is constructed to fit the RTD distribution characteristics of alpine meadows, and the model equation is:


(15) $$R\left(z\right)=\{\begin{array}{cc} \left[1+A_{1}\ln\left(B_{1}\frac{z}{Z}\right)\right]RTD_{\max} & z<z_{RTD_{\max}}\\ \frac{RTD_{\max}^{2}}{A_{2}.P}\times {\text{e}}^{-\frac{\bar{RTD}.z}{B_{2}.Z}} & z\geq z_{RTD_{\max}} \end{array}$$

where *RTD_max_* is the maximum RTD (tips.cm^-3^); *z* is the soil depth (cm); $z_{RTD_{max}}$ is the location of the maximum RTD (cm); *Z* is the thickness of the rhizosphere (cm); *P* is the proportion of part $z>\;z_{RTD_{max}}$ to the total rhizosphere; $\bar{RTD}$ is the average RTD (tips.cm^-3^); and *A*_1_, *A*_2_, *B*_1_, and *B*_2_ are the coefficients related to the RTD (**Table 4**). Since the experimental site d was not measured at $z>\;z_{RTD_{max}}$ , the parameters *A*_1_ and *B*_1_ were estimated by choosing the mean values of the remaining three positions, and the resulting fitted curves showed the same good performance.

**Table 4:** Through the established Eq. 15, the RTD on soil profiles in Exp. 1~4 is fitted.

Sites		*A*_1_	*B*_1_	*R* ^2^	*RMSE*	*A*_2_	*B*_2_	*R* ^2^	*RMSE*
a	Exp. 1	0.65	2.08	0.92	1.69	10.11	3.3	0.73	1.68
	Exp. 2	0.22	2.2	0.98	0.20	8.6	3.5	0.64	1.43
	Exp. 3	0.7	2.3	0.99	0.25	11.9	3.71	0.84	1.32
	Exp. 4	0.18	2.32	0.99	0.05	10.21	4.45	0.96	0.66
b	Exp. 1	0.42	1.89	0.78	1.70	12.95	4.13	0.59	1.53
	Exp. 2	0.19	2.2	0.98	0.21	10.61	4.01	0.86	1.04
	Exp. 3	0.67	1.92	0.81	2.62	15	5.34	0.95	1.03
	Exp. 4	0.15	2.29	0.99	0.09	10.27	4.41	0.99	0.66
c	Exp. 1	0.52	1.85	0.74	2.41	13.38	4.26	0.8	1.37
	Exp. 2	0.2	2.2	0.98	0.20	12.44	5.99	0.9	0.93
	Exp. 3	0.59	2.13	0.95	1.20	16.66	5.54	0.81	1.22
	Exp. 4	0.36	2.56	0.94	0.69	8.43	3.15	0.95	0.87
d	Exp. 1	0.53	1.94			11.02	2.75	0.92	1.02
	Exp. 2	0.2	2.2			9.82	2.92	0.85	1.00
	Exp. 3	0.65	2.12			12.95	3.51	0.81	1.55
	Exp. 4	0.23	2.39			8.46	3.46	0.9	0.57

The parameters R^2^ and root mean square error (RMSE) were obtained. Among them d due to the lack of shallower data, the average values of the other three locations are selected to estimate the values of their coefficients A_1_ and B_1_.

We evaluated the simulation performance of the established root tip density distribution equation by *R* ^2^ and RMSE (**Eq 14**) (**Table 4**), and a large number of results showed that *R* ^2^ was larger and RMSE was less than 2. Therefore, we concluded that the equation could describe the distribution characteristics of RTD in alpine meadows more accurately.

For the description of the RLD, we adopted the more commonly used equation for the RLD distribution as the exponential equation (Feng et al., 2008). Moreover, in this paper, we set the expression of the equation as:


(16) $$R\left(z\right)=C.RLD_{\max}.e^{-D\frac{z}{Z}}$$

where *C* and *D* are the coefficients related to RLD; *RLD_max_* is the maximum RLD (cm.cm^-3^). The equation of the RLD function at each location was obtained by fitting the RLD characteristics of the alpine meadow according to **Eq 16** with the fitted function graph (**Figure 3**). This equation can accurately reflect the distribution of RLD in most cases and also showed excellent performance in this study.

### 3.2 Validation and performance evaluation of the RWU model for alpine meadows

Based on Eqs. 3–10 and Eqs. 15–16, we constructed an alpine meadow RWU model with RTD distribution characteristics and RLD distribution characteristics as key RWU indicators, respectively. The accuracy of the RWU model is usually verified by using a soil water movement model that includes RWU (Eq. 11), simulating the changes in SWC, and comparing the differences between simulated and observed values of SWC.

Due to the variable climate and frequent atmospheric precipitation in the study area, the long time span will lead to significant effects of external factors on SWC, which will make great errors in the model validation process. Therefore, in this paper, we choose the short time span from September 1 to September 4, when the influence of external factors such as rainfall and snowfall in the study area is minimal and the weather conditions are normal and stable, as the starting and ending times for model validation.

Due to the variable climate and frequent atmospheric precipitation in the study area, a long time span will lead to a significant influence of external factors on SWC, which will lead to great errors in the model validation process. Therefore, in this paper, we choose the short time span from September 1 to September 4, when the influence of external factors such as rainfall and snowfall in the study area is minimal and the weather conditions are normal and stable, as the starting and ending times for model validation. At the same time, we use 16 sets of validation results to exclude errors due to chance.

**Figure 4:**
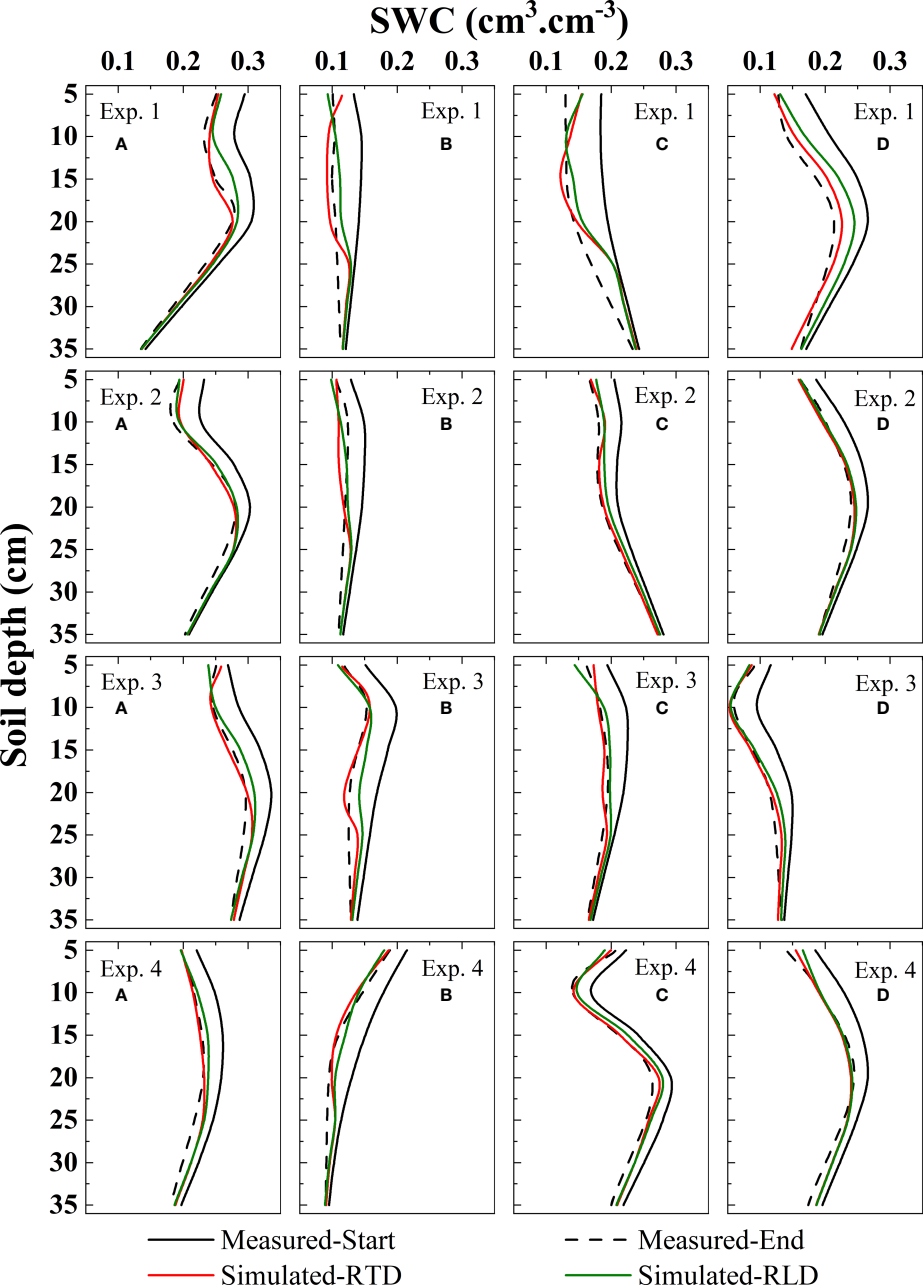
Based on the measured data, the accuracy of the root water uptake models of the sites a, b, c, and d inExp. 1 ~ 4 was verified. It contains soil water content distribution characteristics simulated by root tip density as root water uptake indicator (Simulated-RTD); soil water distribution characteristics simulated by root length density as root water uptake indicator (Simulated-RLD); measured soil water content starting and ending values (Measured-Start and Measured-End).

The model validation results are shown in **Figure 4**. Relatively speaking, the model based on the RTD function simulates the SWC change more realistically. Integrating the simulated and actual values, we obtained the actual and simulated values of SWC change. It was found that both models underestimated the RWU capacity to some extent. Among them, the model based on RTD underestimated 14.93% on average and the model based on RLD underestimated 30.98% on average relative to the actual amount of SWC variation. Comparing the RWU simulated by the two models, we found that the RWU simulated by the model based on RTD increased by 24.64% on average compared to the model based on RLD.

**Figure 5:**
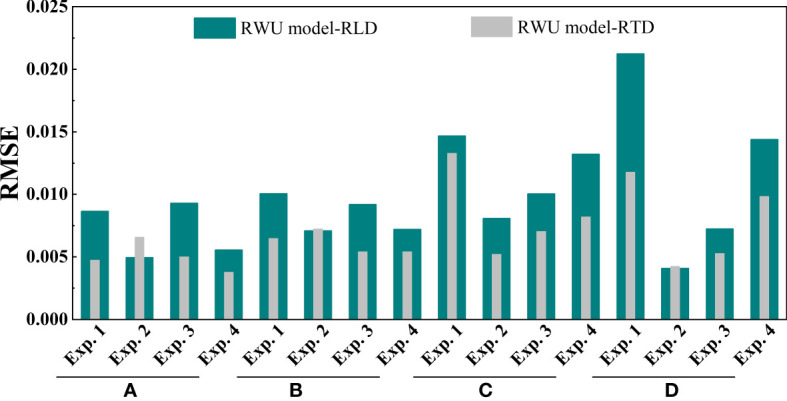
The model simulation performance was evaluated by the root mean square error (RMSE) (Eq. 14). In the process of model validation, we obtained the soil water content (SWC) changes simulated by the model built with root length density (RWU model-RLD) and root tip density (RWU model-RTD) at the sites a, b, c, and d in Exp. 1 ~ 4. Moreover, the simulated SWC distribution was analyzed and compared with the measured SWC distribution by RMSE.

We compared the simulation performance of the two models by RMSE (**Figure 5**). It can be seen that the RMSE obtained from the model based on RTD is low, and it decreases by 40.56% on average compared to the model built on RLD.

Based on the above results, we concluded that the simulation performance of the established root uptake model with root tip density as the key root uptake parameter was high.

Based on the above results, we believe that the RWU model established with RTD as the key water uptake parameter has a high simulation performance.

### 3.3 Distribution characteristics of the RWU rate in alpine meadows

Since the simulation of the RWU model established by RTD is more accurate, this paper analyzes the RWU characteristics by simulating the RWU rate on the soil profile based on the above research method.

**Figure 6:**
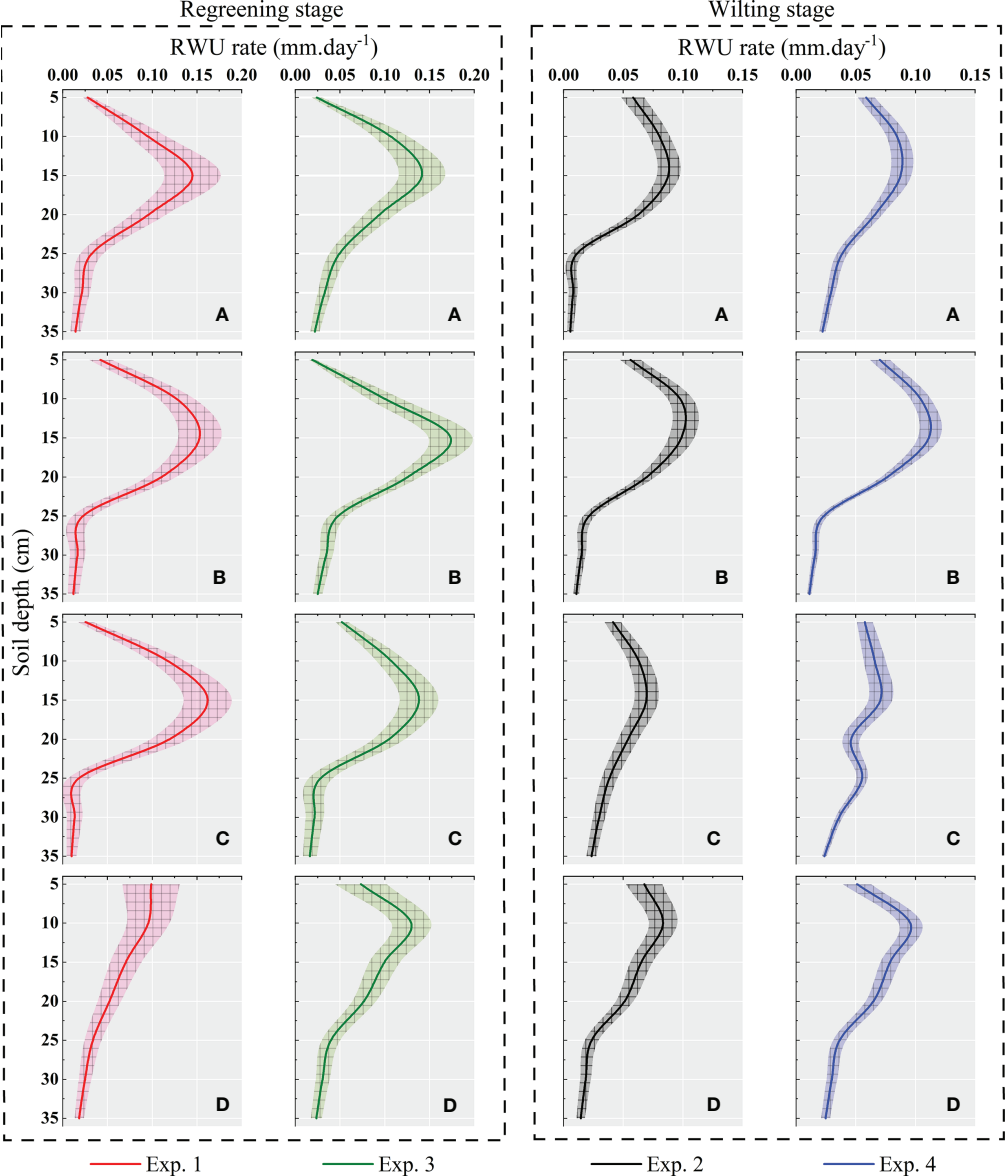
The distribution of root water uptake rate on the soil profile simulated by the root uptake model based on root tip density in Exp. 1 ~ 4 at the sites a, b, c, and d. The root water uptake characteristics at each experimental site during the rejuvenation and wilting stages were also compared.

The daily average RWU rates at different locations at different experimental times are shown in **Figure 6**. It can be found that the characteristics of RWU in alpine meadows have obvious regularity in time and space. In the temporal scale, the RWU rate of the alpine meadow was larger in the regreening stage, and compared with the wilting stage, the RWU rate increased by 22.79%~45.8% in the regreening stage, with an average increase of 30.24%.

At the spatial scale, the intensity of RWU in the soil profile showed a maximum value in the middle rhizosphere, showing a “>“ type change, which is increasing first and then decreasing. At the same time, the RWU rate in the soil profile showed a greater variation in the regreening stage and a flatter variation in the wilting stage. Among them, the top 67% of the roots in the rhizosphere bore an average of 86.76% of the total RWU.

## 4 Discussion

### 4.1 Analysis of the causes of root growth characteristics and root water uptake characteristics

There are obvious spatial and temporal distribution regularities in the root distribution characteristics and RWU distribution characteristics of alpine meadows. Based on the distribution of both the soil profile and different phenological stages, we analyzed the reasons for this regularity.

The pattern of alpine meadow RWU rate in time and space is consistent with the distribution pattern of meadow RTD, which indicates that the intensity of RWU is directly related to the density of the root index that can effectively absorb water. The alpine meadow exhibited a clear regreening stage in which both the root density index and the RWU rate were greater than that of the wilting stage, which is more consistent with the results of scholarly studies on alpine meadows (Wang et al. 2022). The reason for this phenomenon is attributed to the difference in water requirements of plants in different phenological stages.

Since there is a paucity of studies on RWU in alpine meadows, this paper is based on the study of root growth characteristics and water consumption capacity of maize and other (Yu et al., 2015; Zheng et al., 2022) crops as an analogy to alpine meadows. The results showed that the water consumption of plants differed in different phenological stages, with the maximum water consumption in the early stage of plant growth and decreasing in the maturity stage. The water consumption of alpine meadows showed a similar pattern. In the early stage of meadow growth, which is the most vigorous period of meadow growth, the larger growth water demand corresponds to the faster RWU rate. At the end of the meadow growth period, which is the wilting stage, the growth rate of the meadow is slower and the RWU rate is also slow. As a direct factor affecting RWU, the root density index tends to be larger when plant water consumption is higher.

### 4.2 Analysis of model validity based on soil physicochemical properties

In this paper, the accuracy of the RWU model was verified by using the soil water movement equation including RWU, and the simulation performance was compared when RTD and RLD were used as the root index that can effectively absorb water, respectively. The results showed that the simulation performance of the RWU model based on the RTD function was better and the simulated soil water distribution was more consistent with the actual situation. However, there are still some differences between the simulated results and the actual situation. Therefore, we analyzed the correlation between RWU rate, root density index, and soil physicochemical properties to further verify the validity of the model.

**Figure 7:**
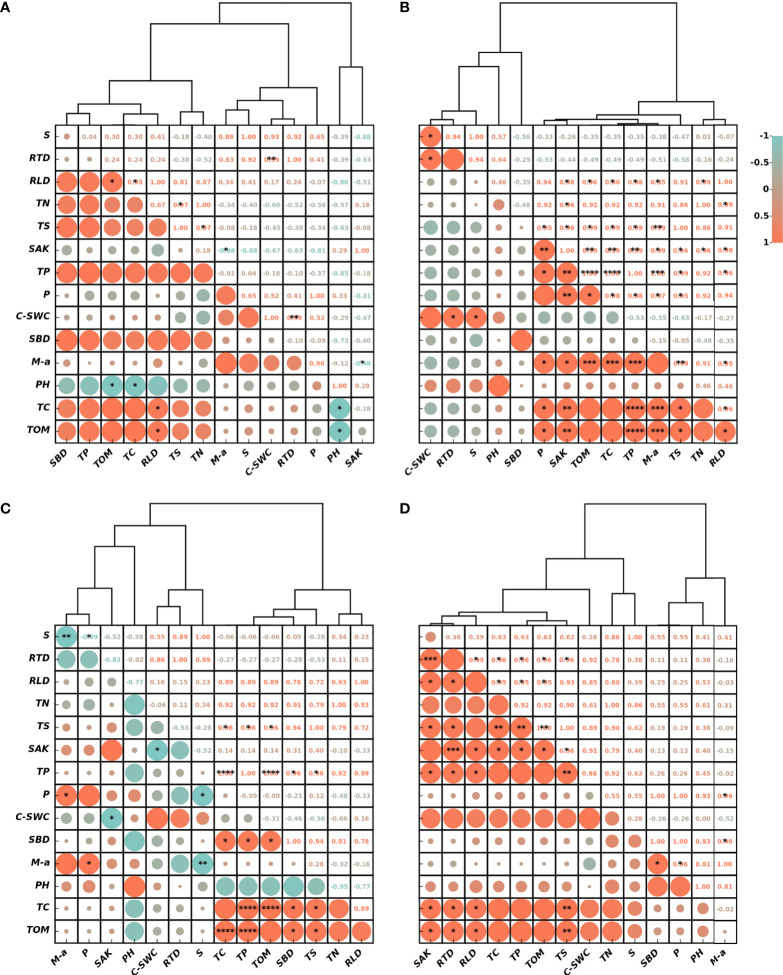
Cluster analysis profiles between root water uptake rate, root density indicators, and soil physicochemical properties in alpine meadows at the sites a, b, c and d **(A–D)**. It contains root water uptake rate (S), root tip density (RTD), root length density (RLD), total nitrogen (TN), total salt (TS), fast-acting potassium (SAK), total phosphorus (TP), porosity (P), soil water change rate (C-SWC), bulk weight (SBD), percentage of macroaggregates (M-a), acidity (PH), total carbon (TC), and total organic matter (TOM). (* means P<0.05, ** means P<0.01, *** means P<0.001, **** means significant level of P<0.0001. The figure was drawn on the https://www.chiplot.online/).

The correlation and cluster analysis plots for the four experimental sites are shown in **Figure 7**. All showed a significant correlation between RWU rate and RTD. The RWU will lead to certain changes in the water content of the soil profile, and the significant positive correlation between the rate of change in SWC and RTD further proves that RTD is the index of effective water-absorbing roots.

There is a clear correlation between RLD and soil physical properties, as the RLD index reflects the strength of root penetration ability (Liu et al., 2020). The higher the porosity of the soil during root growth, the lower the resistance to root growth and the greater the RLD tends to be (Reichert et al., 2009). Also, a correlation was shown between RLD and soil chemical properties, probably because the greater the number of aging decayed roots in areas with higher RLD, the more nutrients from the root decay process are released into the soil, improving the content of many substances such as soil carbon, nitrogen, and phosphorus. However, the correlation between RLD and the rate of change in SWC and the rate of RWU was not significant, which also indicates that RLD is not a key root index affecting RWU and therefore has relatively poor performance in the simulation process.

### 4.3 Parameter selection and model limitations

The parameters of the RWU model, which are the main external factors affecting RWU, are mainly functional indexes such as soil water potential, soil water diffusion rate, and soil hydraulic conductivity. In this paper, SWC and soil depth are used as the independent variables of the model, which are more satisfied with the basic equations of soil hydraulics than the models with increasing time (Su et al., 2017; Liao et al., 2018) as the independent variables of the study, and the soil unsaturated hydraulic conductivity as the parameter of the model is more suitable for the selection of the research focus of this paper. In terms of the dimension of the model, considering that meadows cover the soil surface in the form of a surface, there is a clear difference from the study of individual plants, so this paper chooses to build a one-dimensional model about the soil depth to better represent the RWU of meadow vegetation.

This study also has some limitations in that some of the data calculated for the data were obtained through the literature, and the mean values in the study area did not take into account the variability with specific sites. More studies have utilized the improved Feddes model and thought that soil water potential is a key indicator of RWU rate. In addition, there is a certain interaction mechanism between soil water and solute movement and solute transformation and it has some influence on the accuracy of the mode (Zeng et al., 2018). There is a lack of basic RWU studies in alpine meadows on the QTP, and none of these factors were considered in the development of the RWU model applicable to alpine meadows in this paper. This may also be the reason for the large difference between model-simulated values and measured values in the deep region of the root layer.

## 5 Conclusion

The temporal regularity of root density in alpine meadows showed that the RLD and RTD decreased by 16.2% and 14.6%, respectively, during the wilting stage compared to the regreening stage. The spatial regularity was shown in that the RLD showed a gradual decrease from shallow to deep at each experimental site at different altitudes. The RTD increased and then decreased with increasing soil depth at lower altitude locations and was similar to the RLD distribution characteristics at higher altitude sites.

For the study of alpine meadow RWU, RTD was used as the index of root that can effectively absorb water, and its simulation performance was higher and more consistent with the actual situation.

The RWU characteristics of alpine meadows also showed obvious spatial and temporal regularity. Compared with the wilting stage, the RWU rate in the regreening stage increased by 30.24% on average. Meanwhile, the top 67% of the rhizosphere accounted for 86.76% of the total RWU on average.

Although the model has shown good performance in studying alpine meadows, further work is needed to demonstrate its applicability to other types of plants. There are still few studies on the alpine meadow RWU model, reference materials are scarce, and field experiments have more difficulties, so the indirect validation method of SWC variation has been used for model validation. However, the SWC may still be influenced by other unmeasured factors besides root water uptake and there are some errors. Therefore, there is still a need for improvement and innovation in the experimental method, especially in the direct measurement of RWU capacity, so that the RWU law can be directly verified. Since the model showed high susceptibility to the influence of external precipitation in the validation and reduced its simulation accuracy, and we collected root distribution characteristics for only two key phenological stages, the conclusions obtained were valid only for these two stages. Therefore, in the follow-up study, we also need to take into account the ability of external precipitation to influence SWC and collect root distribution characteristics for longer time stages, to improve the model’s understanding of plant transpiration in a long time span.

## Data availability statement

The original contributions presented in the study are included in the article/**Supplementary Material**. Further inquiries can be directed to the corresponding author.

## Author contributions

BW, DY, BD, and HF contributed to the conception and design of the study. BW, DY, and SX supervised the study activities. Acquisition of basic data was done by BD, HF, and ML. BD wrote the manuscript and performed the simulations. BD and HF built the model and applied it. BW, DY, and HW critically reviewed the manuscript. All authors contributed to this article and approved this version for submission.

## Funding

This study was supported by the National Natural Science Foundation of China (Nos. 52022110, 51879276), the Second Tibetan Plateau Scientific Expedition and Research Program (STEP) (No. 2019QZKK0207), and the IWHR Research & Development Support Program (No. MK0145B022021).

## Conflict of interest

The authors declare that the research was conducted in the absence of any commercial or financial relationships that could be construed as a potential conflict of interest.

## Publisher’s note

All claims expressed in this article are solely those of the authors and do not necessarily represent those of their affiliated organizations, or those of the publisher, the editors and the reviewers. Any product that may be evaluated in this article, or claim that may be made by its manufacturer, is not guaranteed or endorsed by the publisher.
