# Supplementary material for: Construction of root tip density function and root water uptake characteristics in alpine meadows
Source: Front Plant Sci. 2022 Oct 24;13:918397. doi: 10.3389/fpls.2022.918397 (PMC9638077; doi:10.3389/fpls.2022.918397)
Supplement: Supplementary file 1 [file DataSheet_1.pdf]

1.1 Methods for modeling RWU

1.1.1 Calculation of plant transpiration rate in alpine meadows

In this paper, an existing indirect estimation model that can obtain plant transpiration rates more accurately at the study location is used. That is, a variety of plant state parameters are combined with meteorological parameters to calculate the plant evapotranspiration rate at a specific location.

The alpine meadow evapotranspiration rate depends on the values of crop coefficients and references evapotranspiration (Allen et al., 1998), which are related to each other as:

$$K_c = \frac{ET_c}{ET_0} \tag{1}$$

where: ET_c is the plant evapotranspiration rate (mm.day^{-1}); ET_0 is the vegetation reference evapotranspiration rate (mm.day^{-1}); k_c is the crop coefficient, and the current study on crop coefficients in alpine meadows shows that there is a relationship between crop coefficients and vegetation cover and meteorological factors (FAN, 2011). Thus, in this study, crop coefficients for two key phenological stages were obtained based on prototype field observations combined with the model **(Table 1**).

The calculation of the reference evapotranspiration rate is more complex and needs to take into account the influence of a variety of climatic factors. The currently accepted and most widely used standard method for evapotranspiration simulation (Montero et al., 2001; Rouphael and Colla, 2004) is based on the FAO-56-Penman-Monteith (Allen et al., 2006) model that can calculate the reference evapotranspiration rate, which is calculated as:

$$ET_0 = \frac{0.408\Delta\left(R_N - G\right) + \gamma \frac{900}{T+273} U_2\left(e_s - e_a\right)}{\Delta + \gamma\left(1 + 0.34U_2\right)} \tag{2}$$

where G is the sensible heat flux density from the surface to the soil (MJ.m^{-2}.d^{-1}), T means the mean air temperature (℃) at the observed altitude, R_N is the net radiation (MJ.m^{-2}.d^{-1}) of vegetation, U_2 is the wind speed (m.s^{-1}) at the observed altitude, e_s is the saturation vapor pressure (kPa), e_a is the actual vapor pressure (kPa), Δ is the slope of the saturation vapor pressure versus temperature curve (kPa.℃$^{-1}$), γ is the humidity constant (kPa.℃$^{-1}$). In this paper, we obtained the data from three meteorological stations in and around the study area through the National Meteorological Data Center of China, and the locations of the meteorological stations are shown in **Figure 1A**. Based on the ArcGIS10.2 platform, we obtained the specific values of the above meteorological parameters at each experimental site by spatially spreading the differences to each sub-basin.

Plant evapotranspiration can be divided into plant transpiration, canopy interception evaporation,

and soil evapotranspiration (Belmans et al., 1983). The plant transpiration as a part of plant evapotranspiration, their intrinsic relationship is expressed as (Ojha et al., 2009):

$$E_i = 0.0025 \times F_c \times Ri^{0.34} \times T^{0.19} \tag{3}$$

$$E_s = ET_c . f . e^{-c.LAI} \tag{4}$$

$$T_r = ET_c - E_s - E_i \tag{5}$$

where: E_i is the canopy interception evaporation rate (mm.day^{-1}), (Genxu et al., 2012) fitted the canopy interception equation for alpine meadows in their study (**Eq 3**); F_c is the vegetation cover; R_i is the rainfall rate (mm.h^{-1}); E_s is the soil evapotranspiration rate; f and c are regression coefficients, (Belmans et al., 1983) gave accepted soil evapotranspiration estimates in their study, f=1.0 and c=0.6; LAI is the vegetation leaf area index (**Table 1**) and T_r is the plant transpiration rate (mm.day^{-1}). The vegetation transpiration rates at each location in the study area for each experimental stage were obtained by **Eqs 1-5** (**Table 3**).

1.1.2 Calculation of unsaturated hydraulic conductivity of soils

The measurement of unsaturated hydraulic conductivity of soils is difficult, and experimental errors are very likely to occur in experimental measurements. The experimental results obtained are often unsatisfactory. Thus, the method of calculating the unsaturated hydraulic conductivity of soil using the Malen (Mualem, 1976) and Van Genuchten models (van Genuchten, 1980) has been recognized by the majority of scholars because the results are more accurate to reflect the actual situation. The calculation method is as follows:

$$S_e = \frac{\theta - \theta_r}{\theta_s - \theta_r} = \left[1 + (\eta h)^n\right]^{\frac{1}{n} - 1} \tag{6}$$

where: S_e is the effective soil saturation; θ is the measured soil water content (%) (**Table 2**); θ_s is the saturated soil water content (%); θ_r is the residual soil water content (%); h is the soil water potential (m); η and n are empirical constants and combined with the measured values, we obtained η and n for individual experimental site by the inverse method. The unsaturated hydraulic conductivity model for soils (van Genuchten, 1980) was:

$$K = K_s S_e^{0.5} \left[1 - \left(1 - S_e^{\frac{1}{m}}\right)^m\right]^2 \tag{7}$$

where: K is the soil unsaturated hydraulic conductivity (cm.min^{-1}); K_s is the soil saturated hydraulic conductivity (cm.min^{-1}) (Table 2); $m = 1 - 1/n$.

1.1.3 Comparison and selection of RWU models

In this paper, the Selim-Iskandar model (Fred and Molz., 1981) was selected to reflect the alpine meadow RWU:

$$S = \frac{K(h)R(z)}{\int_0^{L_r} K(h)R(z)dz} T_r \tag{8}$$

where: S is the RWU rate (mm.day^{-1}); $R(z)$ is the root density function that can effectively absorb water; L_r is the thickness of the root layer (cm); z is the depth from the ground surface (cm).
